# Dynamic Contrast-Enhanced MRI in the Abdomen of Mice with High Temporal and Spatial Resolution Using Stack-of-Stars Sampling and KWIC Reconstruction

**DOI:** 10.3390/tomography8050178

**Published:** 2022-08-24

**Authors:** Stephen Pickup, Miguel Romanello, Mamta Gupta, Hee Kwon Song, Rong Zhou

**Affiliations:** Department of Radiology, University of Pennsylvania, Philadelphia, PA 19104, USA

**Keywords:** dynamic contrast enhanced, perfusion, radial, stack of stars, respiratory motion

## Abstract

Application of quantitative dynamic contrast-enhanced (DCE) MRI in mouse models of abdominal cancer is challenging due to the effects of RF inhomogeneity, image corruption from rapid respiratory motion and the need for high spatial and temporal resolutions. Here we demonstrate a DCE protocol optimized for such applications. The method consists of three acquisitions: (1) actual flip-angle *B*_1_ mapping, (2) variable flip-angle *T*_1_ mapping and (3) acquisition of the DCE series using a motion-robust radial strategy with *k*-space weighted image contrast (KWIC) reconstruction. All three acquisitions employ spoiled radial imaging with stack-of-stars sampling (SoS) and golden-angle increments between the views. This scheme is shown to minimize artifacts due to respiratory motion while simultaneously facilitating view-sharing image reconstruction for the dynamic series. The method is demonstrated in a genetically engineered mouse model of pancreatic ductal adenocarcinoma and yielded mean perfusion parameters of *K^trans^* = 0.23 ± 0.14 min^−1^ and *v_e_* = 0.31 ± 0.17 (*n* = 22) over a wide range of tumor sizes. The SoS-sampled DCE method is shown to produce artifact-free images with good SNR leading to robust estimation of DCE parameters.

## 1. Introduction

Dynamic contrast-enhanced (DCE) MRI has shown promise as a tool to probe the micro-environment of living tissues. The technique employs rapid imaging methods, typically a spoiled gradient-recalled echo (GRE) acquisition, to observe the changes in image intensity in the tissue of interest during the first pass of a *T*_1_-reducing contrast agent. The method is often employed in cancer diagnosis and treatment monitoring. DCE-MRI is sensitive to changes to the irregular vasculature common in many cancers. Quantitative and semi-quantitative parameters extracted from DCE images have been shown to correlate with therapeutic response in a variety of tumors [1,2,3,4].

Quantitative DCE methods employ least-squares techniques to fit the observed signal changes to a suitable pharmacokinetic model, yielding maps of physiological parameters. The resulting measures of perfusion and/or vascular permeability, notably *K^trans^*, have been shown to correlate with tumor vascularity [5,6] and are useful in the diagnosis and grading of tumors. Quantitative DCE metrics are especially useful in the evaluation of drugs that target the micro-vasculature [7,8,9,10]. Analysis of DCE data typically requires knowledge of the time-dependent concentration of contrast agent in the vasculature feeding the tissue of interest, known as the arterial input function (AIF). In the clinic, the AIF is typically extracted from arteries in the field of view (FOV) of the dynamic images.

The development of oncological therapies benefits significantly from the study of small-animal orthotopically implanted models and genetically engineered mouse (GEM) models of cancer, both of which are more relevant to the human disease than subcutaneous models. Study of these models, in turn, benefits from the availability of diagnostic tools that are similar to those available in the clinic, including DCE-MRI. However, application of DCE-MRI methods in small-animal models presents several significant challenges.

Quantitative analysis DCE data requires an independent map of baseline longitudinal relaxation times for the tissues of interest. The variable flip-angle (VFA) method [11,12] is widely implemented in clinical DCE-MRI protocols due to its acquisition efficiency. However, VFA-based longitudinal relaxation time estimates are known to be very sensitive to radio frequency (RF) inhomogeneity and/or errors in RF power calibration compared to inversion recovery methods [13]. Accurate relaxation time estimation therefore requires an independent measure of the RF field. The Actual Flip-angle Imaging (AFI) method [14] of RF field mapping has the advantage of high acquisition efficiency and this is therefore the method of choice for RF field mapping in compound studies such as DCE.

The combination of high spatial and temporal resolution required for direct observation of the AIF in the blood vessels feeding tumors in murine models is beyond the capabilities of contemporary preclinical instrumentation. In addition, the flow velocities in the great vessels of mice are very high (~1 m/s) relative to the thickness of the imaging slab (~1 cm) typically employed in mouse studies. As such, magnetization in the blood pool does not achieve a steady state before traversing the imaging slab, as is assumed in the modeling. A variety of approaches have been proposed for addressing this limitation, including: use of a fixed parametric model for the AIF [15], use of a population average AIF [16,17], observation of the AIF in the left ventricular blood pool [18], shunting blood to an external catheter in which the AIF is accurately measured [19] and the reference tissue method [20,21,22]. Of these, the reference tissue method has the advantage of providing individualized data directly from the dynamic data without explicitly measuring the AIF.

Cartesian sampled spoiled GRE dynamic MRI of orthotopic tumors of the abdomen of mice suffer from motion artifacts due to rapid respiratory motion. Temporal and spatial resolution are also limited using this approach [23]. Though Cartesian keyhole sampling methods (e.g., TRICKS) have been used to improve temporal resolution, image corruption due to respiration motion persists and some data typically must be excluded from the analysis [24]. Application of respiration gating reduces artifacts but complicates DCE data analysis due to the irregular acquisition timing introduced by the gating. Three-dimensional radial trajectories combined with keyhole/view-sharing techniques have been demonstrated in the context of DCE imaging of xenografts in mice [25,26]. This technique is robust with regard to motion, allows for reconstruction with variable temporal resolution and provides high resolution. However, the 3D radial approach typically requires isotropic resolution, therefore limiting flexibility in tradeoffs between temporal and spatial resolution.

The stack-of-stars (SoS) sampling scheme (radial in two dimensions and phase encoded in the third) has been shown to be effective in mitigating respiratory motion in clinical abdominal and chest DCE studies [27]. Like the 3D radial sampling scheme, the SoS method is robust with regard to motion and is amenable to view-sharing reconstruction techniques. In contrast to the 3D radial technique, it relies on slice selection to achieve localization, making the method well suited for the observation of deep-lying tumors with a homogeneous volume coil.

In the present report, we describe a preclinical DCE protocol that employs SoS sampling in combination with the reference tissue method of data analysis. Though SoS sampling has previously been employed in clinical DCE studies [27] as well as preclinical cardiac studies [28], we believe this to be the first demonstration of the technique in the context of preclinical DCE studies.

## 2. Materials and Methods

### 2.1. Phantom Studies

Five phantoms consisting of sealed 5 mm NMR tubes containing solutions of 18–369 mm MnCl_2_ in deionized water were prepared and used for assessment of the *B*_1_ and *T*_1_ mapping protocols. Longitudinal relaxation times of the phantoms were independently measured on a 9.4 T high-resolution spectrometer (Agilent, Palo Alto, CA, USA) using an inversion recovery pulse sequence with 4 averages (to take advantage of the 4-step phase cycle) and an array of 14 inversion times. Inversion times were scaled such that 3–4 spectra were acquired prior to the nulling point. The recovery time was set to a value greater than 5 times the estimated *T*_1_ (5–15 s). Relaxation measurements were performed at temperatures of 20, 25, 31 and 37 °C and were repeated 3 times on different days for each sample at each temperature. Integrals of peak intensities underwent a three-parameter least-squares fit to an exponential recovery where the adjustable parameters were *M*_0_, *M_z_* (0) and *T*_1_.

For imaging studies, the five phantoms were mounted in a 3D printed support and a temperature probe was taped to the support structure near the center of the sample group. The sample group was positioned in a RF volume coil and a temperature regulated air source was directed through the coil (temperature = 25 °C) after positioning the coil in the magnet. The system was given a minimum of 30 min to achieve thermal stability before performing the SoS-sampled *B*_1_ and *T*_1_ mapping protocols as described in animal scans. Images were reconstructed and *T*_1_ maps were generated both with and without *B*_1_ correction as described below. Mean *T*_1_ values over circular regions of interest (ROIs) were tabulated for each phantom in each slice of the acquired data. Means of the ROI *T*_1_ values were calculated over all slices in the plateau region of the excitation pulse (slices 4–14) and tabulated.

### 2.2. Animal Model

All animal procedures were approved by the institutional animal care and use committee (IACUC) of the University of Pennsylvania (Philadelphia, PA, USA). Animal studies employed a genetically engineered mouse model of pancreatic ductal adenocarcinoma (PDAC) in which Kras and p53 mutations were introduced to the pancreatic epithelium via Cre recombinase [29]. This model, referred to as the KPC mouse, was bred at the Pancreatic Cancer Mouse Hospital of the Abramson Cancer Center of our institute. KPC mice spontaneously developed premalignant Pancreatic Intraepithelial Neoplastic (“PanIN”) lesions at 7–10 weeks of age, leading to invasive PDAC at 17–19 weeks with high penetrance. Tumor screening was undertaken via weekly abdominal palpations starting at 11 weeks of age, followed by ultrasound examination (Vevo 2100, VisualSonics, Toronto, ON, Canada) to estimate the tumor sizes. Mice with confirmed tumor masses (both sexes, 18–25 weeks old) were enrolled in the MRI study. Tumors of size in the range of 80–300 mm^3^ (mean +/− standard deviation = 187 ± 72, *n* = 22) as determined by MRI were enrolled in the DCE study.

### 2.3. MRI

To prepare animals for MRI exams, general anesthesia was induced and maintained via free breathing of 1–3% isoflurane in oxygen through a nose cone. A tail vein catheter was placed with an extension tubing of sufficient length to reach from the magnet isocenter to the end of the magnet bore. The tubing was preloaded with contrast agent in order to minimize dead volume effects. A rectal temperature probe and pneumatic respiration pillow (SAII, Stonybrook, NY, USA) were applied to the animals and the animals were positioned on a 3D printed bed, which was positioned in a 35 × 40 (ID × length) mm quadrature birdcage coil (M2M, Cleveland, OH, USA). The RF coil was positioned in the magnet and regulated warm air source was directed over the animals to maintain core body temperature at 37 °C. Respiration rate and core body temperature were monitored throughout the MRI exam.

All MRI exams were performed on a 9.4 T Avance III console (Bruker, Berillica, MA, USA), equipped with 12 cm ID, 40 G/cm gradients. Following system calibration and generation of scout images, an axially oriented contiguous series of *T*_2_ weighted images spanning the tumor volume was generated using a TurboRare protocol (effective *TE* = 30 ms, *TR* = 1.8 s, echo train length = 8, matrix = 128 × 128, slices = 16, FOV = 32 × 32 mm, thickness = 1.5 mm, averages = 4, total acquisition time ~2 min). This was followed by an axial Actual Flip-angle Imaging (AFI) protocol [14] employing stack-of-stars (SoS) sampling [30,31] (*TE*/*TR*_1_/*TR*_2_ = 1.2/7/35 ms, flip angle = 60°, matrix = 128 × 201 × 16 [read × view × slice], FOV = 32 × 32 × 24 mm, averages = 2, total acquisition time = 8.4 min). The protocol employed a 7 lobe SLR pulse envelope for excitation that was optimized to have minimal out of band excitation. The slab position and orientation, matrix and FOV parameters from the AFI scan were used for all of the subsequent scans. The AFI protocol employed both RF [32] and gradient spoiling to suppress transverse magnetization between repetitions. In phantom studies a spoiling gradient of 16 G/cm amplitude and 3 ms duration was found to be sufficient to fully suppress the residual transverse magnetization. These spoiling parameters were used throughout the animal studies.

Initial animal scans with the AFI protocol yielded images in which the signal from unsaturated blood in the great vessels was an order of magnitude greater than that from the static tissue. This led to streaking artifacts radiating from the vessels in the reconstructed images. The AFI protocol was therefore modified to include outer volume suppression (OVS) to minimize these effects. The OVS consisted of two saturation bands parallel to and on either side of the observation slab. The saturation pulses employed a 90° flip angle and the same pulse envelope and thickness as were used for observation. The addition of OVS resulted in an increased minimal *TR*. The AFI studies that included OVS were therefore performed with *TR*_1_/*TR*_2_ = 13/65 msec and all other parameters identical to those used for the non-OVS scans. The AFI protocol was performed both with and without OVS in order to assess the impact of the OVS on the *B*_1_ map estimates.

The AFI scans were followed by acquisition of variable flip-angle (VFA) *T*_1_ mapping data [11,12] (*TR*/*TE* = 5.5/1.25 ms, flip angles = 2, 5, 8, 12, 16, 20°, averages = 4, total acquisition time ~7 min) using a SoS-sampled spoiled GRE protocol. This was followed by acquisition of the DCE series using a golden-angle [33] SoS-sampled GRE protocol (*TR*/*TE* = 5.5/1.25 ms, flip angle = 9° averages = 1, total views = 8040, total acquisition time = 11.7 min). For the DCE study, baseline data were acquired for 2 min at which time 0.2 mL of Prohance (Bracco Diagnostics Inc., Princeton, NJ, USA) diluted to 10 mm Gd was manually injected in approximately 5 s via the tail vein catheter.

### 2.4. Image Reconstruction and Parameter Map Generation

All image reconstruction and processing were performed using custom codes developed in house in the Interactive Data Language (IDL; Harris Geospatial Solutions, Broomfield, CO, USA) and/or Python. The AFI and VFA data were reconstructed using the identical procedure. First, the data were Fourier transformed and shifted in the slice dimension. The resulting data were then regridded to a 128 × 128 Cartesian array using the Kaiser-Bessel kernel and a convolution window of 4 points. The regridded data were then Fourier transformed in the remaining two dimensions and divided by the deconvolution function to yield the reconstructed images. The dynamic data were reconstructed using a similar approach with the exception that the with *k*-space weighted image contrast (KWIC) method [31] of view-sharing was employed. The KWIC processing employed 3 levels with 50 views encoding the center of *k*-space per time point resulting in 157 time frames and a temporal resolution of 4.3 s.

RF field maps were generated from the AFI images using the relation [14]

(1) $$\alpha =\arccos\frac{rn-1}{n-r}$$

where *n* = *TR*_2_/*TR*_1_ = 5.0, *r* = *S*_1_/*S*_2_, and *S*_1_ and *S*_2_ are the signal intensities acquired with *TR*_1_ and *TR*_2_, respectively. Note the above relation assumes that *TR*_1_, *TR*_2_ << *T*_1_. This condition is well satisfied for the acquisition parameters and tissues of interest (*T*_1_ > 2 s) in the current study.

The flip angle calculated via the above relation was then divided by the nominal flip angle to yield the *B*_1_ correction maps. The resulting maps had missing data points within the animal and background where there is insufficient signal intensity. As the RF field is known to vary smoothly in space, the contribution of these effects to subsequent analysis was minimized by fitting the measured field maps to a smooth function and the resulting fits were used for further analyses. The *B*_1_ correction maps were fit on a slice-by-slice basis to the following low order two-dimensional polynomial using least-squares methods

(2) $$\frac{\alpha \left(x,y\right)}{\alpha_{0}}=b_{3}xy+b_{2}x+b_{1}y+b_{0}$$

where *α* and *α*_0_ are the AFI and nominal flip angles, respectively, *b_i_* are the adjustable parameters, *x* and *y* are the coordinates in the x–y plane ranging from −*N_p_*/2 to *N_p_*/2-1 where *N_p_* is the number of points in the corresponding dimension. The resulting polynomial was then sampled over the entire field of view to generate the field correction maps used in subsequent calculations. Maps of longitudinal relaxation times (*T*_1_) were generated by pixelwise non-linear least-squares fits of the VFA image signal intensity to the Ernst equation

(3) $$S\left(\alpha \right)=M_{0}\frac{\sin\alpha \;\left(1-E_{1}\right)}{\left(1-E_{1}\cos\alpha \right)}$$

where *E*_1_ = $exp\;\left\{-TR/T_{1}\right\}$ and *TR* is the repetition time. The relaxation time and equilibrium magnetization (*M*_0_) were the adjustable parameters and the *M*_0_ term includes the effects of several scaling parameters including *T*_2_ * and receiver gain. The *T*_1_ maps were calculated both with the nominal flip angle and with the corrected flip angle in order to assess the impact of the field map on the *T*_1_ estimates. In cases where multiple *B*_1_ maps were available (i.e., with and without OVS), *T*_1_ maps were calculated for each *B*_1_ map.

Regions of interest (ROI) for tumor, kidney cortex and spinal muscle were manually drawn in the DCE images corresponding to maximum tissue contrast agent concentration for each slice spanning the tumor using ImageJ [34]. The *T*_2_ weighted images and *T*_1_ maps were used to guide the ROI drawing process. The ROIs from different slices where then combined to yield volumes of interest (VOI) for which pixel statistics were generated for the various parameter maps.

Quantitative analysis of the DCE time series was performed for each pixel in the VOIs using the reference tissue method [22,35] with muscle serving as the reference tissue. This analysis began with conversion of the time-dependent pixel intensity, *S*(*t*), to relaxation rate, *R*_1_(t) = 1/*T*_1_ (t), using the relation

(4) $$R_{1}\left(t\right)=-\frac{1}{TR}\ln\left[\frac{A-S\left(t\right)/S_{0}}{A-S\left(t\right)\cos\alpha /S_{0}}\right]$$

where *TR* is the repetition time, *S*_0_ is the signal intensity prior to the bolus arrival, *α* is the flip angle used to acquire the dynamic data, and

(5) $$A=\frac{\left(1-E_{10}cos\alpha \right)}{\left(1-E{\;}_{10}\right)}$$

where $E_{10}=exp\;\left\{-TR/T_{10}\right\}$ and *T*_10_ is the pre-contrast longitudinal relaxation time determined in the VFA experiment. The relaxation rates were then converted to contrast agent concentrations using the relation

(6) $$C\left(t\right)=\frac{R_{1}\left(t\right)-R_{10}}{r_{1}}$$

where *r*_1_ = 4.6 s^−1^ mm^−1^ [36] is the relaxivity of the contrast agent in the tissue. Non-linear least-squares analysis was then used to fit the tissue concentration curves to the relation [22]

(7) $$\frac{d\;C_{TOI}}{dt}=\frac{K_{TOI}^{Trans}}{K_{RR}^{Trans}}\frac{d\;C_{RR}}{dt}+\frac{K_{TOI}^{Trans}}{v_{e,RR}}C_{RR}-\frac{K_{TOI}^{Trans}}{v_{e,TOI}}C_{TOI}$$

where we have introduced the sub-script notation to indicate parameters associated with the tissue of interest (TOI) and reference region (RR), $K_{TOI}^{Trans}$ and $v_{e,TOI}$ are the adjustable parameters and values for the reference tissue parameters in muscle were assumed to be $K_{RR}^{Trans}$ = 0.1 min^−1^ and $v_{e,RR}$ = 0.1 [22]. Color overlays of the fitted parameter maps on the *T*_2_ weighted images were then generated and statistical analysis of the parameter values was performed over the VOIs.

## 3. Results

### 3.1. Phantom Studies

AFI studies of the phantom array were used to determine the spoiler gradient parameters necessary to assure suppression of transverse magnetization between repetitions and assess the accuracy of the *T*_1_ mapping protocol. The AFI implementation employed in the current study uses a loop structure in which the acquisition portion of the sequence is repeated six times with the RF pulse enabled on only the first two passes [37] resulting in an effective *TR* ratio of 5:1. In our implementation, the receiver is enabled for every pass through the loop. The data from the first two passes was used for calculation of the field maps and the remaining data provides a measure of spoiling efficiency. Any signal observed in the third or later passes is due to incomplete spoiling. The spoiler gradient area increased gradually until the signal acquired in the third pass was below noise levels for the phantom array. Complete suppression of signal was achieved with a spoiler applied on the slice axis of duration 3 ms and amplitude 16 g/cm (gradient area: *A_g_* = 480 mT ms/m). This requirement for a large spoiling gradient is consistent with previously published simulated and experimental optimizations of the spoiler gradient for the AFI method [38]. There it was demonstrated that accurate *B*_1_ estimates are achieved independent of RF spoiling phase angle increment with *A_g_* = 459 mT ms/m and a *TR* = 20 ms for samples with *T*_2_ = 37 ms. The modestly larger *A_g_* required here is likely due to the longer transverse relaxation times of the phantoms employed in the current study.

Both the SoS AFI and VFA protocols yielded images of the phantoms that were free of artifacts and had a SNR in the range of 12–46. The estimated *B*_1_ corrections for the phantom studies were very flat in the transverse dimensions with mean values that ranged from 0.95 to 1.01 indicating accurate *B*_1_ calibration. The variation of *B*_1_ correction with respect to slice number reflects the response profile of the RF pulse used for observation, with a broad plateau region that drops off sharply near the edges of the FOV (data not shown). The estimated *B*_1_ correction within the plateau region of the pulse oscillated ±3% with respect to slice number which is somewhat larger than expected based on simulations of the response profile or the RF pulse used for excitation.

**Figure 1:**
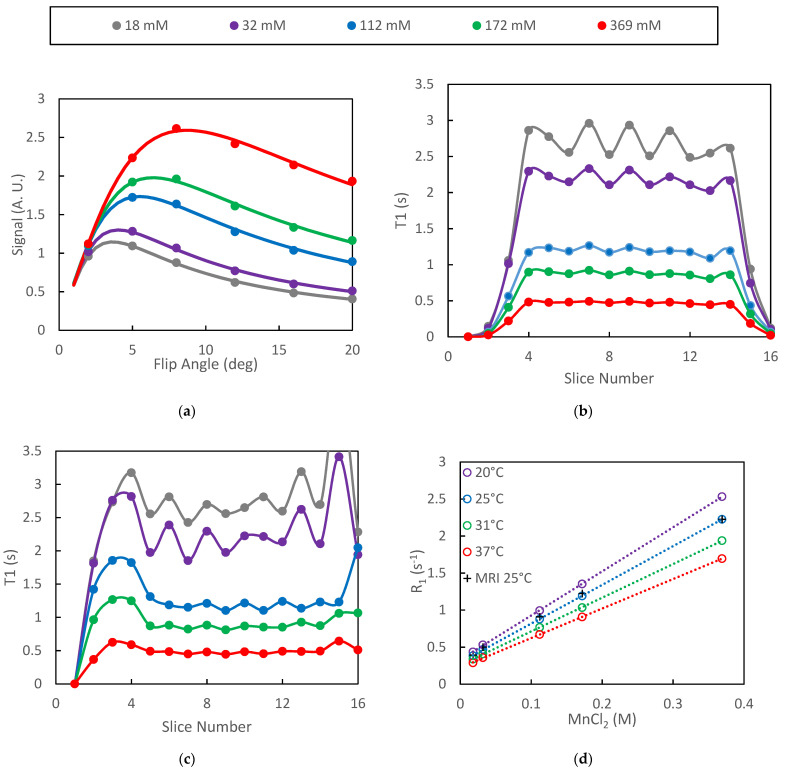
Data from VFA study of MnCl_2_ phantoms are presented: (**a**) ROI signal intensity vs. flip angles is accurately fit by the model; (**b**) oscillations in the mean *T*_1_ with respect to slice number increased with increasing *T*_1_; (**c**) inclusion of *B*_1_ correction increased the width of the *T*_1_ profile as well as amplified oscillations near the edge of the slab; (**d**) relaxation rate plots were highly linear both for the spectroscopic (inversion recovery) and VFA protocols.

Plots of mean signal intensity from the VFA protocol as a function of flip angle were well characterized by least-squares fits to Equation (3) for all of the phantoms (Figure 1a). In the figure, the points are the mean VOI signal intensities averaged over the plateau slices (slices 4–14) and the solid lines are the model fits. Inclusion of *B*_1_ correction did not significantly alter the quality of the fits in the plateau region of the slab as the true/nominal *B*_1_ ratio was approximately 1.0 for those slices. The absence of systematic differences between the model and the measured data is an indication of spoiling efficacy in the VFA protocol [39]. The range of *T*_1_ values represented by the phantoms spans that which is expected for in-vivo DCE studies while the range of *T*_2_ values (28–300 ms) is larger than those expected in-vivo. The optimized spoiling gradient parameters are therefore expected to be adequate for our in-vivo studies.

The signal intensity generated by the VFA protocol was found to oscillate with respect to slice number in the plateau region of the selected slab. This oscillation propagated to the relaxation time estimates and increased in amplitude as *T*_1_ increased (Figure 1b). Inclusion of the *B*_1_ correction did not significantly alter the *T*_1_ estimates in the plateau slices but did extend the number of slices for which *T*_1_ estimates were be generated (Figure 1c).

The spectroscopically determined *T*_1_ estimates generated on different days were highly repeatable with standard deviations of approximately 1% (*n* = 3) of the measured *T*_1_ values. Plots of the spectroscopically determined relaxation rates vs. concentration were very linear (*R*^2^ = 0.999) for all temperatures studied (Figure 1d). Linear regression of the spectroscopic data yielded relaxivities of MnCl_2_ of 5.96, 5.23, 4.55 and 3.99 s^−1^ mm^−1^ for temperatures of 20, 25, 31 and 37 °C, respectively. These results are slightly higher than previously reported values of 5.0 and 3.9 s^−1^ mm^−1^ at 20 and 37 °C, respectively [40]. The relaxation rates as determined by the SoS-VFA protocol fall directly over those determined spectroscopically at 25 °C (Figure 1d) therefore demonstrating the accuracy afforded by the VFA technique.

### 3.2. Animal Studies

**Figure 2:**
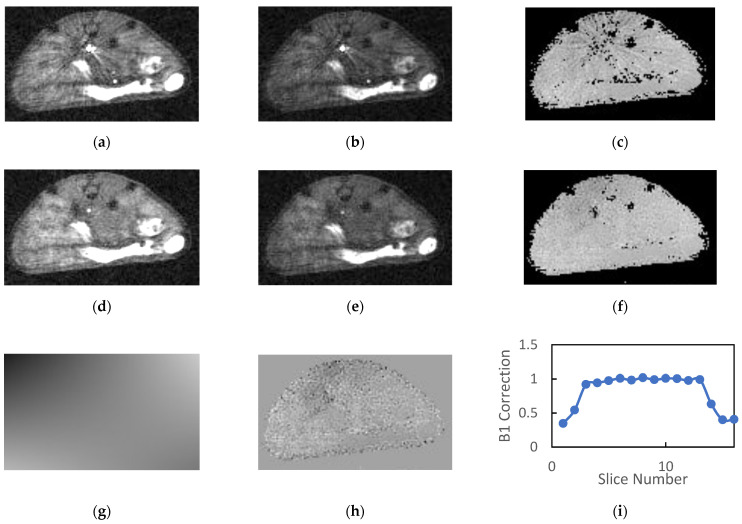
Typical images generated by the SoS AFI protocol are presented: (**a**) *TR*_1_ and (**b**) *TR*_2_ images generated without OVS exhibit streaking artifacts that propagate to the (**c**) *B*_1_ maps. The addition of OVS reduces the intensity of the blood signal in the great vessels for the (**d**) *TR*_1_ and (**e**) *TR*_2_ images therefore eliminating the previously observed streaking artifacts in the (**f**) *B*_1_ maps. The (**g**) fits of the *B*_1_ maps to a low order 2D polynomial exhibited no systematic errors and had (**h**) low homogeneous residues. (**i**) Plots of the normalized *B*_1_ estimates versus slice number reflect the product of the RF excitation profile and the longitudinal sensitivity of the coil.

A total of 22 animals were examined, of which 19 were scanned with AFI both with and without OVS. The animals tolerated the 30–40 min protocol well with no adverse events and recovered from anesthesia quickly. Typical AFI images generated without OVS and their corresponding *B*_1_ maps are shown in Figure 2a–c. The *TR*_1_ and *TR*_2_ images in the figure have approximately equal windowing and are free of respiratory motion artifacts. The mean SNR (*n* = 19) for muscle/kidney/tumor was 7.6/9.3/7.9 and 7.4/8.7/7.3 for the *TR*_1_ and *TR*_2_ images, respectively. If windowed such that the background level is visible, there is clearly artifactual signal in the background regions of the images which contributes the noise estimates and reduces the reported SNR values. As such these values represent a lower limit for the SNR.

As noted previously, the reconstructed AFI images exhibited a large dynamic range due to the combination of large flip angle, short *TR* and inflowing unsaturated blood in the great vessels. The blood in the great vessels typically had an intensity that is 5–10 times greater than that of the static tissue. When these images were windowed appropriately for observation of the tissues, streaking artifacts radiating from the great vessels were observed in the reconstructed images as is evident in Figure 2a–c.

The calculated field maps were very flat within each slice indicating the high degree of transverse homogeneity of the RF volume coil. However, the streaking artifacts present in the source images were somewhat amplified in the calculated *B*_1_ maps (Figure 2c). The mean *B*_1_ correction values in plateau region of the slab (slices 4–13) was consistently close to but greater than unity (1.07 ± 0.04, *n* = 19).

Inclusion of OVS in the AFI protocol significantly reduced the signal intensity of blood in the great vessels and eliminated the streaking artifacts in both the source images and the calculated *B*_1_ maps (Figure 2d–f). The mean SNR (*n* = 19) for muscle/kidney/tumor was 11.0/13.2/11.5 and 10.9/12.1/10.3 for the *TR*_1_ and *TR*_2_ images, respectively. This represents a 37 to 45% gain in SNR relative to images acquired without OVS.

The 2D polynomial fits of the field maps provided a very accurate model of the calculated *B*_1_ field (Figure 2g). Note the figure is windowed to accentuate spatial variation. The magnitude of the spatial variation is actually quite small relative to the homogeneous component indicating a high degree of homogeneity in the transverse dimensions. This is also indicated by the fact that the mean values for the high order parameters in the polynomial fit were consistently two or more orders of magnitude smaller than the zero-order parameter (*b*_0_). Mean residues between the *B*_1_ correction maps and their polynomial fits were approximately 1% of the *B*_1_ correction and had minimal spatial variation for all slices within the plateau region of the slab (Figure 2h).

Plots of mean normalized *B*_1_ vs. slice number (Figure 2i) were flat for the center 8–10 slices and dropped off precipitously at the edges of the imaging slab. These plots reflect the product of the slice profile and the axial variation in RF coil sensitivity. Note the slab thickness was set to 85% of the FOV in the slice direction in order to minimize wrap around effects in the slice dimension. The inclusion of OVS and longer *TR* employed in the OVS studies had a minimal impact on the mean *B*_1_ estimates in the plateau region of the slab. The mean *B*_1_ correction with outer volume saturation (*n* = 19) was 1.10 ± 0.05 and ranged from 0.95 to 1.14.

**Figure 3:**
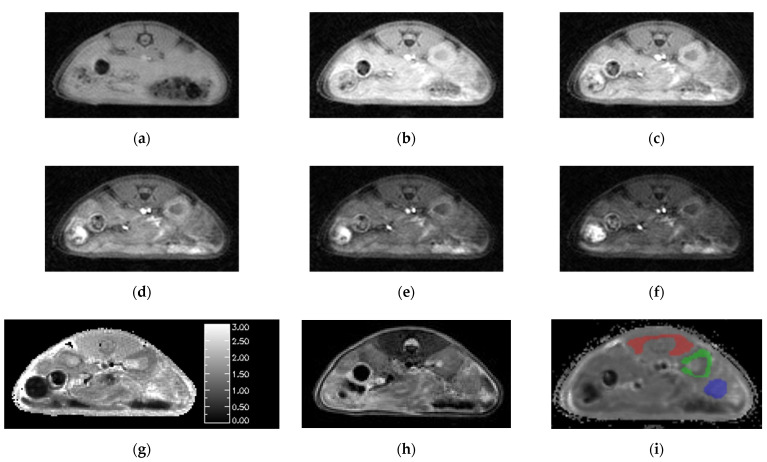
Images generated by the SoS-VFA protocol for flip angles of (**a**) 2, (**b**) 5, (**c**) 8, (**d**) 12, (**e**) 16 and (**f**) 20° were free of motion, had high SNR and yielded high quality (**g**) *T*_1_ maps. The (**h**) *T*_2_ weighted images provided good soft tissue contrast. VOI analysis was performed for three tissue types: (**i**) muscle (red), kidney (green) and tumor (blue).

Animal studies using the SoS-VFA *T*_1_ mapping protocol yielded images free of respiratory motion artifacts (Figure 3a–f). The images in the figure use identical windowing in order to highlight the flip-angle dependence of the signal intensity. There was no need to employ OVS in the VFA protocol due to the use of significantly lower flip angles than those employed in the AFI protocol. The mean SNR for the VFA images acquired with flip = 8° for the three tissues of interest was muscle/kidney/tumor = 23.3/30.2/25.5. We use 8° flip data for SNR estimation in the VFA protocol because it facilitates comparison to the dynamic data which are acquired with a comparable flip angle. The least-squares fits to Equation (3) were free from systematic errors and yielded high quality *T*_1_ maps (Figure 3g).

The *T*_2_ weighted images provided good resolution and high contrast between tumor and healthy tissues (Figure 3h). However, slight mis-registration between the *T*_2_ weighted and SoS images in the slice dimension were occasionally observed which motivated use of the DCE images for ROI definition. Boundaries for the ROIs were readily identified by simultaneously viewing the *T*_1_ maps and *T*_2_ weighted images. The resulting ROIs for muscle, kidney cortex and tumor are overlayed on one of the dynamic images from the corresponding study (Figure 3i).

**Figure 4:**
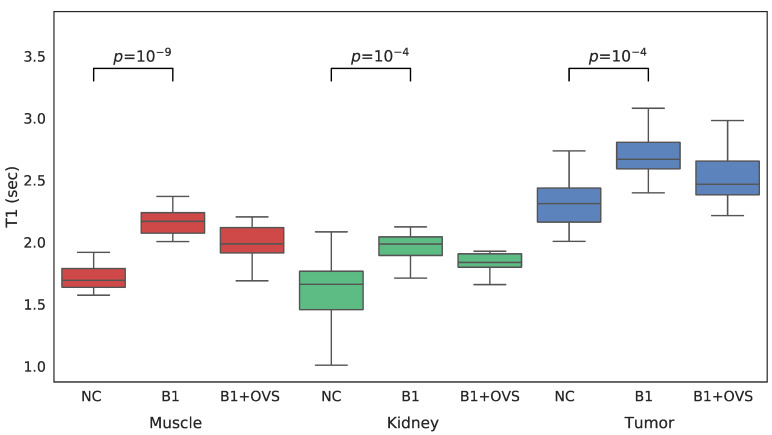
Whisker plots of SoS-VFA *T*_1_ estimates generated without *B*_1_ correction (NC), with *B*_1_ correction but no OVS (B1) and with *B*_1_ correction and OVS enabled (B1 + OVS) for muscle (left, red), kidney (center, green) and tumor (right, blue).

For each VFA data set acquired both with and without OVS, *T*_1_ maps were generated three different ways in order to assess the impact of the various parameter estimation methods. *T*_1_ maps were calculated: (1) without *B*_1_ correction, (2) with *B*_1_ correction, and (3) with *B*_1_ correction that employed OVS. A comparison between the three approaches to estimating *T*_1_ is shown in the whisker plots of Figure 4 for each of the VOIs of interest. Mean *T*_1_ and standard deviations are also summarized in Table 1. The addition of *B*_1_ correction resulted in a statistically significant increase in the estimated *T*_1_ value for all three tissue types (*t*-test *p* less than 0.0001) while the addition of OVS did not significantly alter the *T*_1_ estimates (*t*-test *p* = 0.849/0.503/0.256 muscle/kidney/tumor) or their standard deviation. The addition of *B*_1_ correction to the *T*_1_ analysis is believed to provide more accurate *T*_1_ values since the *B*_1_ correction factor of ~1.07 was highly reproducible. The *T*_1_ estimates generated using the *B*_1_ correction with OVS enabled were therefore used to analyze the dynamic data.

**Figure 5:**
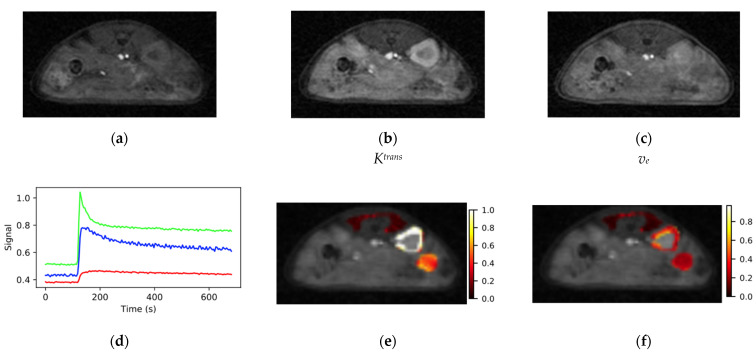
Typical images from the SoS-DCE protocol (**a**) before bolus arrival, (**b**) at the bolus peak and (**c**) during washout are shown. (**d**) VOI response curves for muscle (red), kidney (green) and tumor (blue) had high SNR and the expected form. Maps of (**e**) *K^trans^* and (**f**) *v_e_* were elevated at the tumor periphery relative to the core. Color bar scales are min^−1^ for *K^trans^* and are unitless for *v_e_*.

Representative images from the SoS-DCE protocol prior to bolus arrival (**a**), at the bolus peak (**b**) and during washout (**c**) are shown in Figure 5. Identical windowing was employed each image in the figure. The images are of high SNR and free from motion artifacts. Prior to bolus arrival, mean (*n* = 22) image SNR for the tissues of interest was muscle/kidney/tumor = 9.4/11.6/9.9. Minimal degradation in image quality relative to the AFI and VFA data was evident due to the use of the KWIC sampling/view-sharing scheme [31]. Though not evident in the figure, streaking artifacts were occasionally observed in the images corresponding the maximum in the bolus due to the large dynamic range between the signal in the great vessels and the surrounding tissue.

Plots of VOI mean signal intensity vs. time for the three tissues of interest from the dynamic series had high SNR and the expected time dependence. The signal from the highly perfused kidney cortex (green, Figure 5d) consistently rose from baseline to its maximum in approximately 10 s (2–3 frames) and had a sharp peak. The peak in the kidney signal was followed by a rapid signal decline resembling an exponential decay to approximately 60% of the peak intensity with a time constant of ~20 s. The remaining signal washed out with a rate on the order of several minutes. The tissue response curve for muscle (red, Figure 5d) exhibited a much slower wash-in phase than kidney with typical time to maximum on the order of 60 s and the observed change in signal intensity was on the order of 20% of that observed for kidney. The form of the muscle and kidney tissue response curves were highly reproducible between voxels within the VOI and between animals.

The tissue response curves for tumor (blue, Figure 5d) were more variable both within a given tumor and between animals than those observed for normal tissues. The time to maximum for the tumor signal fell between those observed for kidney and muscle. Maps of *K^trans^* and *v_e_* were heterogeneous within the tumor (Figure 5e,f) with elevated levels around the periphery relative to the tumor core. Mean values (*n* = 22) of *K^trans^* and *v_e_* were 0.74 ± 0.19 min^−1^ (mean ± standard deviation) and 0.38 ± 0.14 for kidney and 0.21 ± 0.12 min^−1^ and 0.31 ± 0.17 for tumor relative to reference muscle *K^trans^* and *v_e_* set at 0.1 min^−1^ and 0.1, respectively.

## 4. Discussion

We have demonstrated a DCE-MRI protocol suitable for observation of deep-lying tumors in the abdomen of mice. The protocol consists of three scans, specifically: (1) *B*_1_ mapping with actual flip-angle imaging, (2) variable flip angle *T*_1_ mapping and (3) the dynamic series. All three scans employ a stack-of-stars sampled spoiled gradient echo acquisition. This sampling scheme has multiple advantages in the context of DCE imaging. The over sampling of the center of *k*-space afforded by SoS sampling makes the scan robust with regard to corruption by respiratory motion. In addition, the sampling scheme is amenable to view-sharing reconstruction techniques such as KWIC [31] which allow tradeoffs between spatial and temporal resolution to be made during image reconstruction of the dynamic data. Our method is a direct translation of the previously demonstrated clinical technique for DCE in the chest to abdominal studies in mice [27] without the use of self-gated respiratory phase binning for motion compensation. The motion correction methods used in the clinical study are not feasible in small animals because the interval between consecutive samples of the center of *k*-space is long relative to the respiration period in small animals. The present study relies on the oversampling of the center of k-space and fact that the magnitude of the respiration motion is significantly reduced in the abdomen of anesthetized animals relative to that in the chest.

Small-animal cardiac studies employing SoS sampling combined with navigator echoes have also been demonstrated. This method relies on performing multiple repetitions and using the navigator data to partition the acquired data into different cardiac phases. This approach motion compensation does not translate to the current study due to the single pass nature of the DCE protocol. 

One limitation of the radial sampling scheme is that reconstruction of data sets with a large dynamic range often results in streaking artifacts. The combination of large flip angle and inflow of unsaturated blood in the great vessels resulted in such artifacts in the AFI protocol. We have demonstrated that these artifacts are readily eliminated by application of OVS techniques.

The impact of *B*_1_ correction, both with and without OVS, on the *T*_1_ estimates was also investigated. The corrections were minimal in phantom studies and had an insignificant impact on the *T*_1_ estimates. However, in the animal studies the AFI measurements revealed a consistent underestimation of the RF power by approximately 7% in the plateau region of the excitation pulse. The magnitude of this underestimation was not significantly affected by the addition of OVS. However, the addition of OVS did improve the SNR and overall image quality in the AFI scans. A failure to account for the underestimation of RF power was shown to result in an underestimation of *T*_1_ in the VFA study. For the equipment used in the current study, the under estimation of RF power appears to be due to inaccuracy in the RF power calibration routine. As such it is possible that use of an alternate power calibration method may eliminate the need for the *B*_1_ mapping protocol therefore reducing the total acquisition time of the DCE exam.

The combination of SoS sampling and KWIC image reconstruction yielded high-resolution images (matrix = 128 × 128 × 16) with a temporal resolution of 4.3 s with minimal reduction in image quality relative to fully sampled data. The resulting tissue response curves were well characterized by the reference tissue model. Maps of *K^trans^* and *v_e_* were relatively homogeneous within kidney cortex while a pattern of elevated *K^trans^* in tumor periphery relative to the core was observed in many KPC tumors.

Several DCE studies of the abdominal organs in mice have been previously reported including examination of pancreas [41,42,43,44], placenta [45,46], kidney [24,47] and liver [2,48,49,50,51]. Most of these studies employed full Cartesian sampling for acquisition of the dynamic data. The combined temporal and spatial resolution that can be achieved in the dynamic series with this approach is significantly lower than that presented in the current study. Improvements in resolution for Cartesian sampled DCE data may be achieved by application of keyhole acquisition methods [24] or compresses sensing image reconstruction [52,53]. However, the resulting dynamic data are often corrupted by motion artifacts. A variety of approaches for minimization of motion artifacts have also been proposed including securing the tissue of interest with a plastic platen [41], prospective gating [18], non-affine image registration [42] and use of navigator echoes [49]. Though application of these methods generally reduces the frequency of occurance of motion artifacts, some data corruptoin persists.

A limited number of authors have taken advantage of the motion robustness of radial samping for acqusition of abdominal DCE data in mouse models. Both radial 3D gradient echo (3D-GRE) [48] and ultra-short echo time (UTE) [47] methods have been used to generate dynaimc data. These techniques provide robustness to motioin artifacts that is comparable to the metods presented in the current report. In addition, these methods provide higher temporal and spatial resoltuion than can be achieved with Cartesian sampling when combined with view-sharing image reconstruction methods. The UTE method has the added advantage of minimizing *T*_2_ * contributions to the observed signal changes. However, the SoS sampling scheme employed in the current study requires fewer views per image than the radial 3D GRE and the UTE methods. SoS sampling also provides greater flexibility with regard to extent and resolution in the slice dimension. This flexibility can be used to minimize the extent of the acquired data to include only the tissue of interest. These factors in turn result in higher temporal and/or spatial resolution provided by the SoS scheme than can be achieved with the 3D radial methods for a given TR.

The results presented in here highlight both the advantages and the limitations of the radial sampling techniques in the context of dynamic imaging. As is evident in the images presented, motion artifacts in radially sampled data do not manifest as coherent ghosts as is the case Cartesian sampling. Less evident in the data presented is the fact that image corruption due to motion as well as *B*_0_ inhomogeneity is present in the images in the form of incoherent artifacts spread throughout the images. Here it was noted that such artifacts interfered with our SNR estimates. Simultaneously, the lack of systematic errors between the observed data and the models employed suggests that such artifacts do not significantly interfere with the tissue signals. Further improvements in image quality may be achieved using self-gating techniques to eliminate views that are corrupted by motion and through the use of artificial intelligence image reconstruction techniques to correct for the effects of *B*_0_ inhomogeneity.

## 5. Conclusions

We have demonstrated a DCE-MRI method that uses golden-angle SoS sampling throughout with KWIC image reconstruction for the dynamic data. This is the first time this method of acquisition of DCE data has been demonstrated in small-animal models. The resulting protocol provides high spatial (matrix = 128 × 128 × 16) and temporal (4.3 s) resolution and minimal sensitivity to motion artifacts. The technique was demonstrated in an orthotopic mouse model of pancreatic ductal adenocarcinoma, and the resulting data were analyzed using the reference tissue method. The resulting method was shown to provide high-resolution maps of *K^trans^* and *v_e_* in this model.

## Figures and Tables

**Table 1:** Mean and standard deviations of *T*_1_ estimates (s) for the volumes of interest as generated using three different methods.

Method	Muscle	Kidney	Tumor
No *B*_1_ Correction	1.70 ± 0.11	1.60 ± 0.14	2.27 ± 0.19
*B*_1_ Correction w/o OVS	2.14 ± 0.08	1.93 ± 0.12	2.69 ± 0.21
*B*_1_ Correction with OVS	2.16 ± 0.07	1.87 ± 0.10	2.56 ± 0.19

## Data Availability

Data supporting the results of this study will be deposited at the GitHub open-source platform (https://pennpancreaticcancerimagingresource.github.io/data.html (accessed on 6 May 2022)) upon the publication of the paper.
